# Bottom-up and top-down effects on phytoplankton communities in two freshwater lakes

**DOI:** 10.1371/journal.pone.0231357

**Published:** 2020-04-09

**Authors:** Yanran Li, Jiao Meng, Chao Zhang, Shuping Ji, Qiang Kong, Renqing Wang, Jian Liu

**Affiliations:** 1 Environment Research Institute, Shandong University, Qingdao, China; 2 College of Geography and Environment, Shandong Normal University, Jinan, China; 3 School of Life Sciences, Shandong University, Qingdao, China; Qingdao Agricultural University, CHINA

## Abstract

The relative importance of bottom-up versus top-down effects in aquatic ecosystems remains a longstanding and ongoing controversy. To investigate these effects on phytoplankton communities in freshwater lakes, phytoplankton and zooplankton were sampled, and physical-chemical variables were measured during spring and summer in two important freshwater lakes in northern China: Nansi Lake and Dongping Lake. The redundancy analysis results showed that phytoplankton density and biomass were regulated by physical-chemical variables (bottom-up effects) and predation (top-down effects) together, and the former was more prominent in both lakes. However, the correlation analysis indicated that the top-down effects of zooplankton on phytoplankton were not significant in spring and summer in both lakes, while the bottom-up regulation of physical-chemical variables on phytoplankton had different patterns in the two lakes. In Nansi Lake, the bottom-up effects of physical-chemical variables on phytoplankton were weaker in summer than that in spring due to the abundant nutrients in summer. In Dongping Lake, the bottom-up effects of physical-chemical on phytoplankton were significant both in spring and summer, and the dominant bottom-up control factor shifted from total nitrogen in spring to total phosphorus in summer, with an increased ratio of nitrogen to phosphorus due to changes in limiting factors. In the two studied lakes, with fish culture, the bottom-up effects of phytoplankton on zooplankton were more important than the top-down effects of zooplankton on phytoplankton. These results demonstrate the interactions between phytoplankton and zooplankton and highlight the importance of phytoplankton regulation in freshwater lakes, which has implications for the effective management of freshwater lake ecosystems.

## Introduction

Phytoplankton and zooplankton not only play important roles in aquatic ecosystems but also serve as key indicators for water quality assessment [1–4]. Phytoplankton is the primary producer in lake ecosystems, producing oxygen and organic matter through photosynthesis. Zooplankton is a predator of phytoplankton, connecting primary producers with more advanced consumers in the biological chain and providing a crucial link in the aquatic food web. Seasonal changes in plankton communities have been found [5], which are performed not only as changes in species number, density, biomass and diversity but also as seasonal changes in community structure [6–8]. Moreover, the dominant species of phytoplankton may also change between different seasons within a year [9]. These changes are mainly influenced by physical-chemical (bottom-up effects) and predation (top-down effects) through the aquatic food web [10,11].

The bottom-up effect means that a lower trophic level in the biological network affects the community structure of higher trophic levels by means of resource restriction [12]. The top-down effect refers to a higher trophic level influences the community structure of a lower trophic level through predation [10]. McQueen et al. [13] proposed that the bottom-up effect is strongest at the bottom of the food web and weakens further up the trophic levels, while the top-down effect is strongest at the top of the food web and weakens further down the trophic levels. Some studies have indicated that nutrients influence the density and species composition of phytoplankton through bottom-up effects while predation by zooplankton (top-down effects) controlled the size, distribution, and abundance of phytoplankton [14–16]. Zhang et al. [17] and Song et al. [18] found that phytoplankton was more influenced by the bottom-up effects of environmental factors, while Severiano et al. [19] indicated that the top-down effects of zooplankton had a more significant influence on phytoplankton. Experimental studies have suggested that phytoplankton is controlled more by the bottom-up effects of nutrients than the top-down effects of zooplankton when zooplankton are under strong predation pressure from fish [20,21]. However, few studies have addressed the seasonal dynamics of bottom-up and top-down effects, which have an important influence on phytoplankton succession in freshwater [22]. It is necessary to explore the bottom-up and top-down effects in phytoplankton regulation and the seasonal dynamics of these influences, as the dynamics and structure of phytoplankton communities play an important role in aquatic ecosystems. Understanding the influence of bottom-up and top-down effects in freshwater lakes would provide meaningful evidence for better management.

Nansi Lake and Dongping Lake are two major freshwater lakes in Shandong Province in northern China that are important diversion lakes on the eastern route of the South-to-North Water Diversion Project. In preparation for the water diversion project, water quality and biodiversity of both lakes were restored while they were used for economic development, such as aquaculture. Therefore, these two lakes are suitable for studying the different responses of phytoplankton and zooplankton in lakes in which water quality has been improving in recent years. Based on the measurement of physical-chemical variables, phytoplankton and zooplankton in Nansi Lake and Dongping Lake, we tested the following hypotheses: (i) the bottom-up effects of physical-chemical have more significant regulatory effects on phytoplankton than the top-down effects of zooplankton, and (ii) different patterns in the seasonal regulation of bottom-up and top-down effects exist in different lakes.

## Materials and methods

### Ethics statement

No specific permissions were required to collect the samples in our study. We confirm that the field studies did not involve endangered or protected species.

### Study area and sampling sites

**Fig 1:**
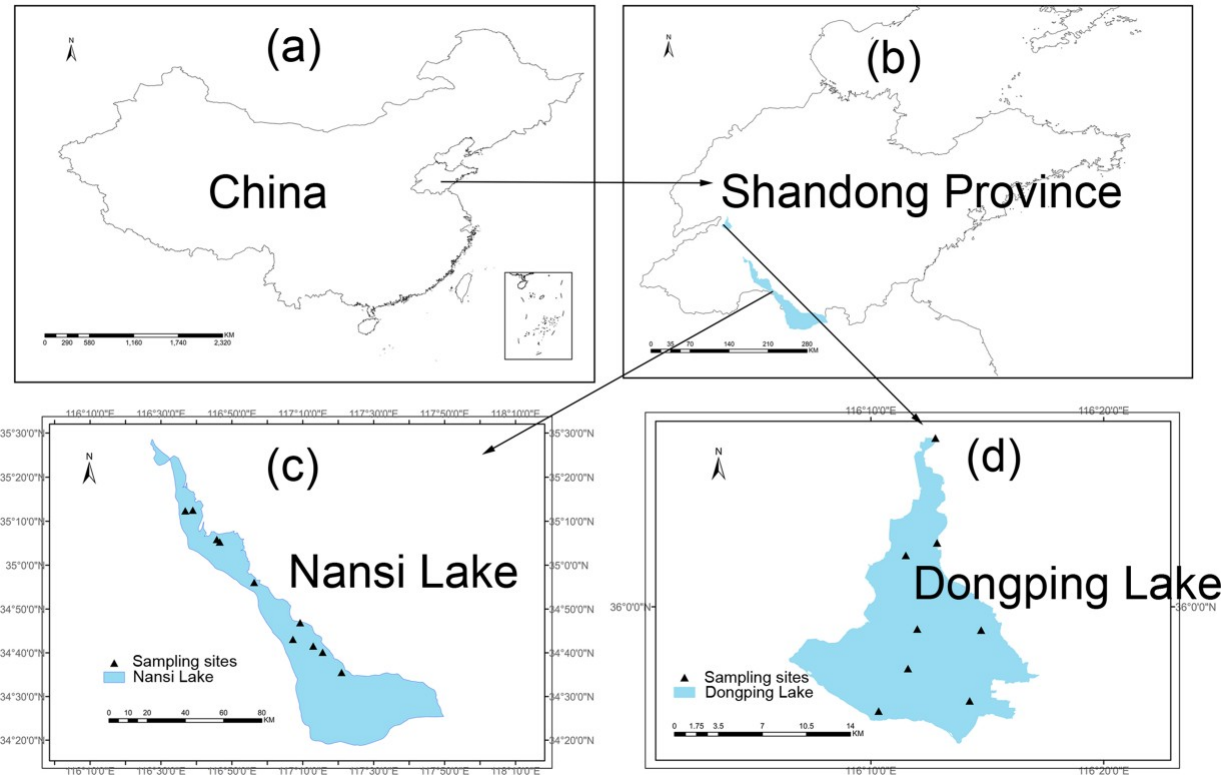
Location and sampling sites of Nansi Lake and Dongping Lake, China. (Software of ArcGIS 10.2 and Adobe Photoshop CS6 were used in drawing the figure. The outline of study area and two lakes was drawn by using ArcGIS (version 10.2) and referring to the map from http://www.dsac.cn/).

Nansi Lake (116° 34’ E– 117° 21’ E, 34° 27’ N– 35° 20’ N) is the largest freshwater lake in northern China, with a total area of 1266 km^2^. The lake consists of four connected lakes, which are Nanyang, Dushan, Zhaoyang and Weishan. There are about fifty-three inflowing rivers and three outflowing rivers. Nansi Lake plays a vital role in the east route of the South-to-North Water Diversion Project, which is the largest project to solve the demand for water resources in northern China. With the warm-temperate monsoon climate, Nansi Lake has an annual temperature of 13.7°C and a total capacity of 6.37×109 m^3^. Dongping Lake (116° 00’ E– 116° 30’ E, 35° 30’ N– 36° 20’ N) is located in Tai’an in the middle of Shandong Province, China. The total area of Dongping Lake is 632 km^2^, and the average water depth of the lake is 2.5 m. Dongping Lake is the catchment for the tributaries of Dahan River in the Yellow River Basin. It is also one of the storage lakes on the east route of the South-to-North Water Diversion Project, and it also has a warm-temperate monsoon climate. Well distributed sampling sites were established across Nansi Lake (n = 10) and Dongping Lake (n = 8) (Fig 1), and samples were collected in spring (April and May) and summer (July and August) from both lakes in 2015.

### Sampling methods

Plankton samples were collected at each site with three replications by two different sized plankton nets, with mesh sizes of 112 and 64 μm. The qualitative samples, which were collected by the plankton nets with mesh sizes of 112 μm (macro plankton samples) and 64 μm (micro plankton samples), were classified under a light microscope [23,24]. The quantitative samples were collected using different approaches. Micro plankton samples, which were collected with a 1 L plankton sampler, were kept in brown bottles and fixed with Lugol’s iodine solution for microscopic enumeration. Macro plankton samples, which were collected with 40 L water samples and concentrated to 30 mL through a plankton net with a mesh size of 112 μm, were fixed with formaldehyde solution for microscopic enumeration. The biomass and density of plankton were calculated following the Handbook of fishery natural resource investigation in the inside water area [25]. The biomass calculations, which used the specific gravity and body length-weight regression equation, were converted, taking sample volume into account.

Water samples were collected along with biodiversity samples *in situ* at a depth of 0.5 m using a 1 L Ruttner water sampler. There were three replications for each sample and the samples were carried back to the laboratory as soon as possible under low-temperature conditions for total nitrogen (TN) and total phosphorus (TP) measurement using a 5B-3B(V8) multi-parameter water quality meter and LH-3BNT total nitrogen analyzer. Water temperature (WT) and chlorophyll a were measured in the field using a thermometer and a portable chlorophyll a meter. Dissolved oxygen, pH and water transparency were also measured in the field using a portable dissolved oxygen meter, a pH meter and a Secchi disk. Chemical oxygen demand (COD_Cr_) was measured in the laboratory using 5B-1 COD rapid monitor, and chemiluminescence detection of permanganate index (COD_Mn_) and NH3-N were measured using acidic potassium permanganate method and spectrophotometry.

### Statistical analysis

Redundancy analysis (RDA) was used to test the variables with a significant top-down or bottom-up influence on phytoplankton in Nansi Lake and Dongping Lake as the detrended correspondence analysis (DCA) revealed that the gradient length of the response data was less than three. In addition, Pearson correlations among phytoplankton density, phytoplankton biomass, different physical-chemical variables, zooplankton (especially Crustacea) density, and zooplankton biomass were performed comparing spring and summer periods. The RDA highlighted the influence of water temperature, TN, and TP on phytoplankton. Therefore, correlation analysis among these variables was performed for further study. To explore the top-down effects, correlation analysis between phytoplankton and zooplankton was performed across the seasonal factor and biotic indices of density and biomass. Four different patterns were assessed: the same indices for phytoplankton and zooplankton in the same season, the same indices for phytoplankton and zooplankton in different seasons, different indices for phytoplankton and zooplankton in the same season, and different indices for phytoplankton and zooplankton in different seasons. SPSS 22.0 and CANOCO for windows were used to implement the aforementioned analyses.

## Results

### Abiotic and biological variables in Nansi and Dongping lakes

**Fig 2:**
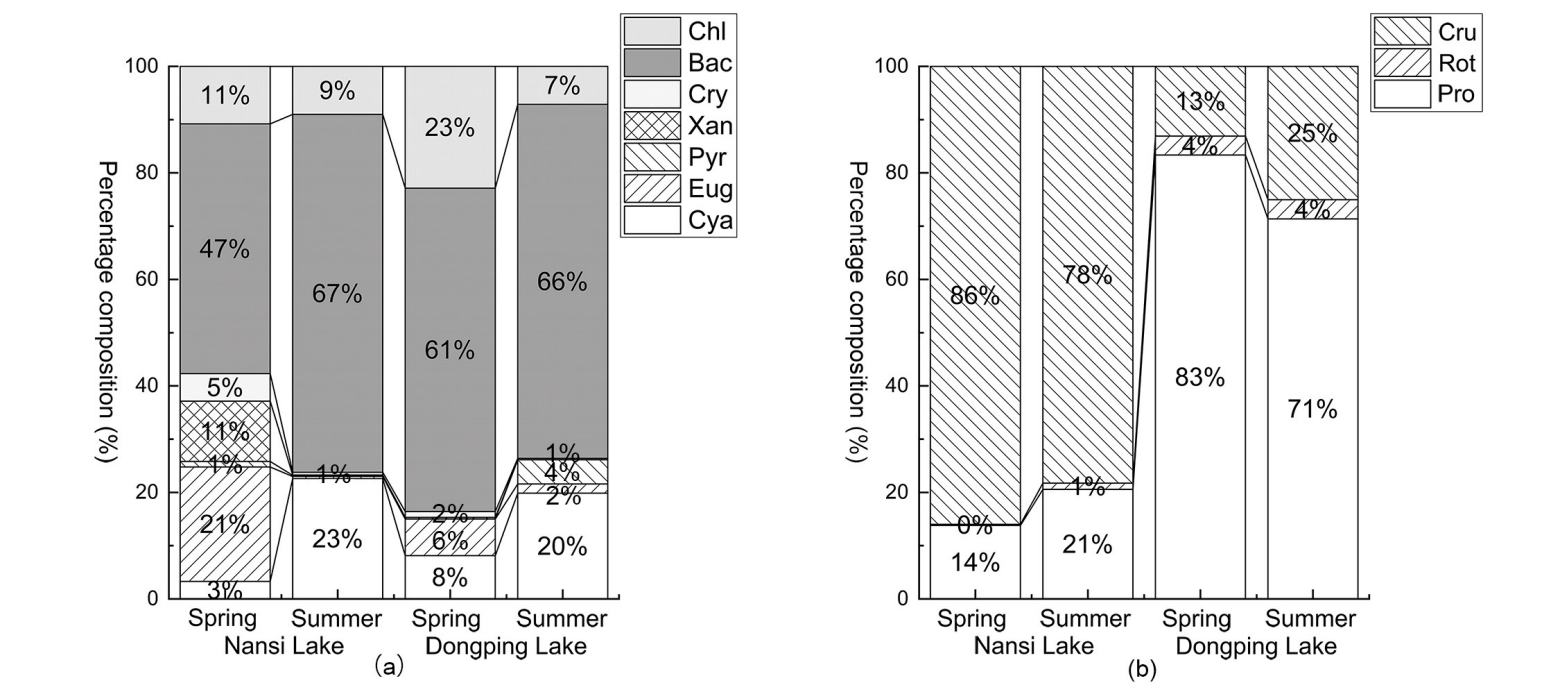
Percentage composition of phytoplankton (a) and zooplankton (b). Phytoplankton taxa are Chlorophyta (Chl), Bacillariophyta (Bac), Cryptophyta (Cry), Xanthophyta (Xan), Pyrrophyta (Pyr), Euglenophyta (Eug), Cyanophyta (Cya); zooplankton taxa are Crustacea (Cru), Rotifera (Rot), Protozoa (Pro).

In 2015, the average water temperature in summer increased by about 12°C compared with that in spring (Table 1), with decreased transparency and dissolved oxygen compared with that in spring. Water quality deteriorated in summer in both Nansi and Dongping lakes, with changes in different indices. The concentrations of chlorophyll a, TP, TN, COD_Mn_, and COD_Cr_ increased in Nansi Lake in summer, while the concentrations of chlorophyll a, TN, and COD_Mn_ increased in Dongping Lake in summer. The density and biomass of phytoplankton and the density of zooplankton were higher in summer than that in spring in both lakes. However, the biomass of zooplankton increased in summer in Dongping Lake, whereas, the biomass of zooplankton showed a higher value in spring in Nansi Lake. The dominant algae in the two lakes were Bacillariophyta with seasonal changes in their composition in spring and summer (Fig 2). The sub-dominant algae in Nansi Lake changed from Euglenophyta in spring to Cyanophyta in summer. In Dongping Lake, the sub-dominant algae changed from Chlorophyta in spring to Cyanophyta in summer. For zooplankton, Crustacea was dominant in Nansi Lake in both spring and summer, while Protozoa was the dominant in Dongping Lake.

**Table 1:** Physical-chemical variables in Nansi Lake and Dongping Lake in 2015 in different seasons.

Variable	Nansi Lake		Dongping Lake	
	Spring	Summer	Spring	Summer
Water temperature (°C)	19.9±1.06	30.86±1.52	17.39±2.95	30.98±0.76
pH	8.29±0.91	8.16±0.45	8.54±0.65	8.45±0.50
Water transparency (m)	0.54±0.33	0.19±0.13	0.58±0.36	0.36±0.18
Dissolved oxygen (mg·L^-1^)	10.57±1.69	5.96±1.97	9.81±1.59	8.35±1.74
Chlorophyll a (mg·L^-1^)	12.23±6.52	23.63±12.25	9.97±6.85	27.81±26.43
Total phosphorus (mg·L^-1^)	0.04±0.02	0.10±0.04	0.18±0.21	0.14±0.08
Permanganate index (mg·L^-1^)	5.17±1.20	5.59±1.33	4.89±0.75	5.67±1.01
Chemical oxygen demand (mg·L^-1^)	20.25±9.89	32.51±7.77	34.61±21.07	28.03±7.10
NH3-N (mg·L^-1^)	0.75±0.20	0.36±0.28	0.48±0.27	0.20±0.08
Total nitrogen (mg·L^-1^)	1.99±1.86	2.81±1.82	0.93±0.38	1.35±0.50

### The response of phytoplankton variables to bottom-up and top-down effects

**Fig 3:**
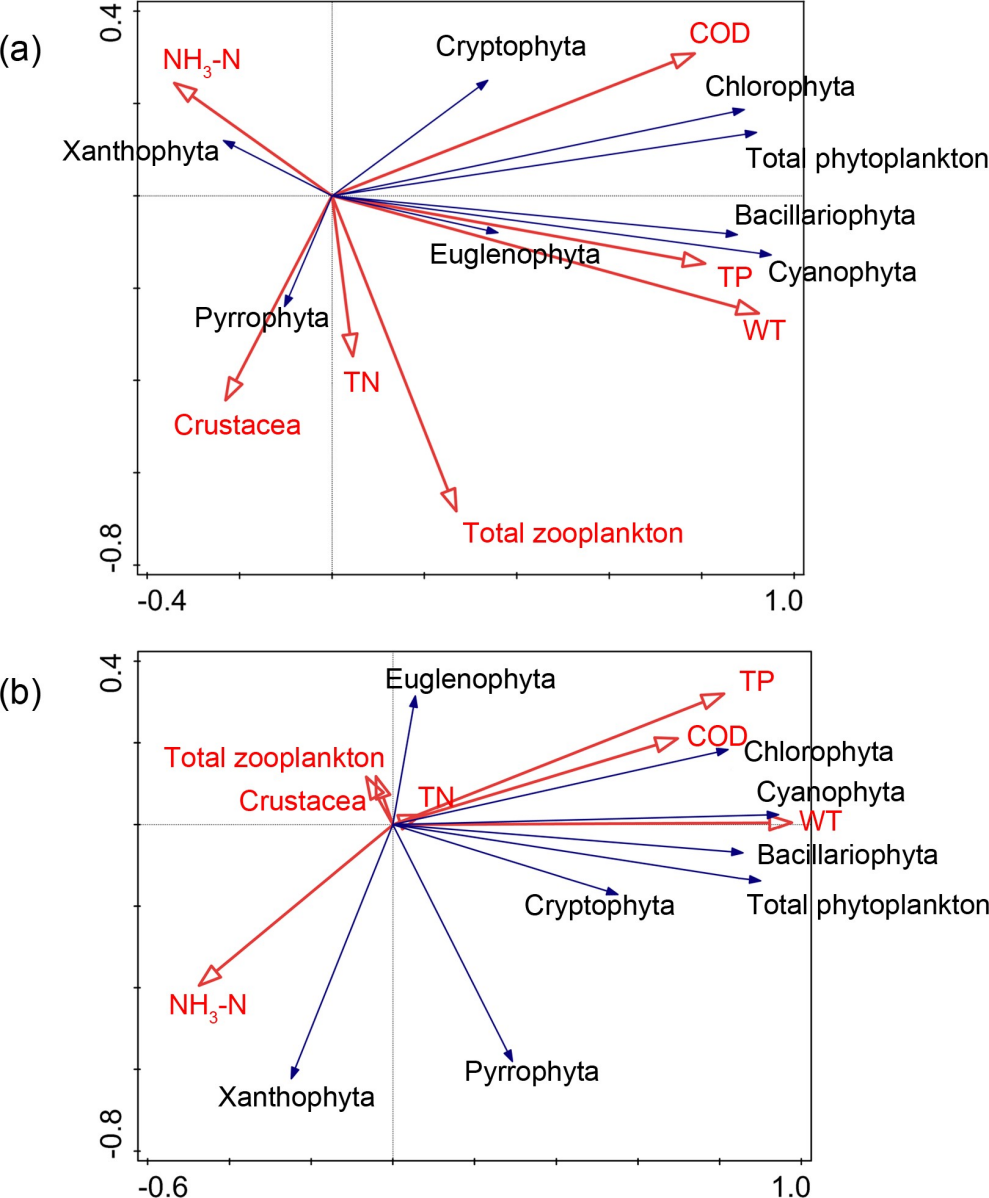
Redundancy analysis (RDA) plots for different phytoplankton phyla, zooplankton and water physicochemical parameters in Nansi Lake. ((a) density; (b) biomass).

The RDA (Fig 3A) demonstrated the ranking of phytoplankton density, zooplankton density, and physical-chemical factors in Nansi Lake, of which the first two axes accounted for 69.72%. Euglenophyta density and Cyanophyta density showed a positive correlation with TP. In addition, Euglenophyta density was positively correlated with water temperature, while Pyrrophyta density was positively influenced by Crustacea density. There were no significant correlations between the density of all phyla of phytoplankton in Nansi Lake and the densities of zooplankton and Crustacea. For biomass, the RDA ranking of phytoplankton biomass, zooplankton biomass, and physical-chemical factors in Nansi Lake showed that the first two axes accounted for 58.24% of the total variation (Fig 3B). The biomass of phyla of phytoplankton was mainly related to physical-chemical variables, and the Cyanophyta biomass presented a significant positive relationship with water temperature. The biomass of Pyrrophyta was significantly affected by the biomass of zooplankton and Crustacea, indicating the predation effects on Pyrrophyta in Nansi Lake.

**Fig 4:**
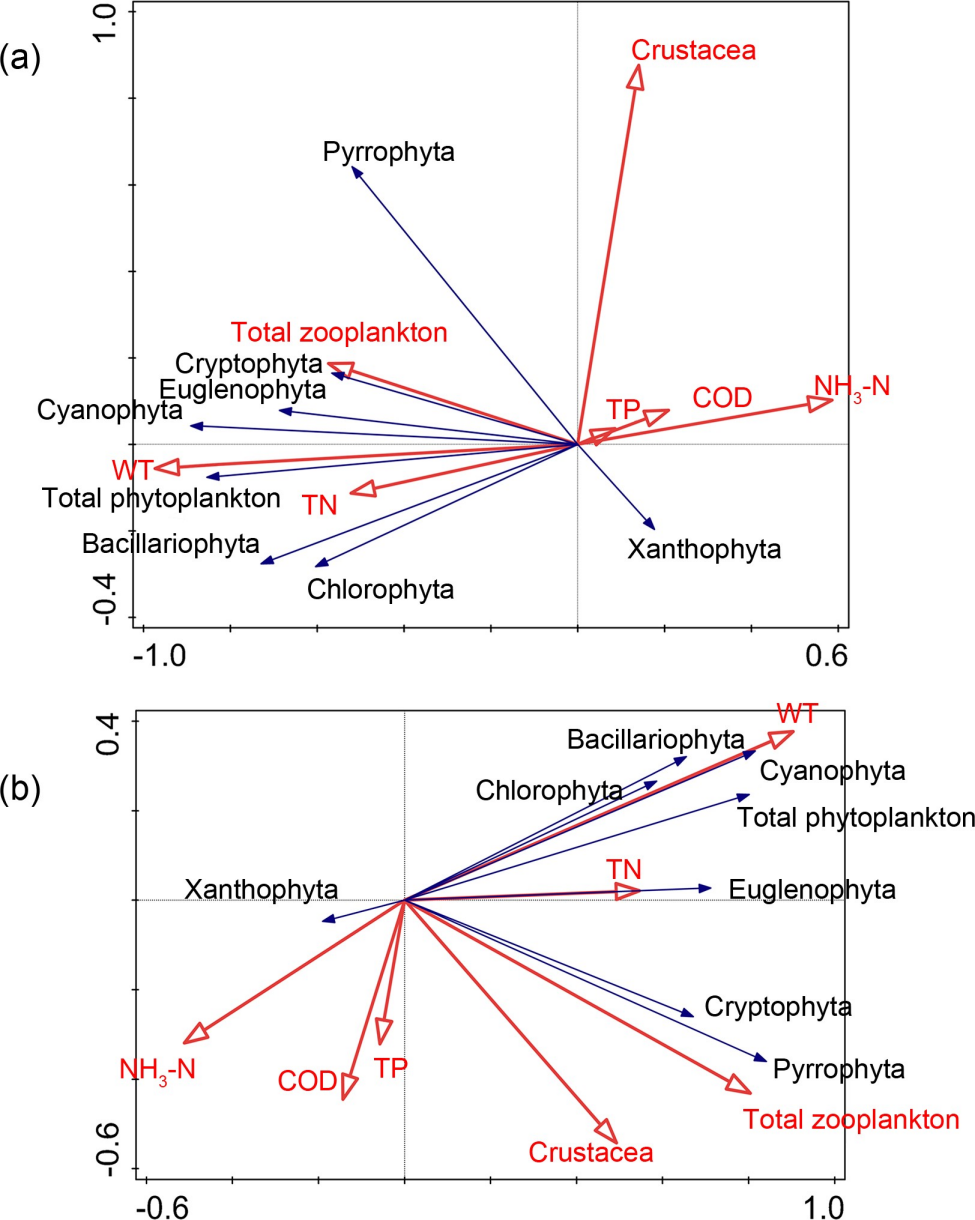
Redundancy analysis (RDA) plots for different phytoplankton phyla, zooplankton and water physicochemical parameters in Dongping Lake. ((a) density; (b) biomass).

The RDA results for the phytoplankton variables and physical-chemical factors in Dongping Lake showed that the first two axes accounted for 63.82% of the total variation in phytoplankton density (Fig 4A) and 64.32% of the total variation in phytoplankton biomass (Fig 4B). The density and biomass of phytoplankton were mainly related to water variables that differed with phylum and physical-chemical factors. There was no significant correlation between phytoplankton and zooplankton in Dongping Lake, suggesting that weak top-down effects of zooplankton and Crustacea existed.

### Seasonal patterns of bottom-up and top-down effects

The relationships between water quality variables and phytoplankton density and biomass differed between spring and summer in Nansi and Dongping lakes (Tables 2 and 3). In Nansi Lake in spring, the densities of Euglenophyta and Bacillariophyta were positively related to TP, and the biomass of Euglenophyta was positively influenced by water temperature and TN. The phytoplankton showed no significant relationship with water temperature, TP, or TN in summer in Nansi Lake. However, there were different patterns in the bottom-up effects of physical-chemical on phytoplankton in Dongping Lake during seasonal changes. Most of the phytoplankton, except Cyanophyta, Pyrrophyta, and Chlorophyta, were significantly influenced by water temperature and TN in spring, and most of the phytoplankton, except Pyrrophyta and Xanthophyta, were influenced by TP in summer in Dongping Lake. These seasonal changes showed the strong bottom-up effects of physical-chemical on phytoplankton in Dongping Lake in both spring and summer.

**Table 2:** The Pearson correlations between water temperature, total phosphorus, total nitrogen and phytoplankton of Nansi Lake in different seasons.

	Spring			Summer		
	WT	TN	TP	WT	TN	TP
Density_TPh_	-0.499	-0.286	-0.233	0.541	-0.183	0.222
Density_Cya_	0.325	-0.406	0.313	0.6	-0.149	0.294
Density_Eug_	0.246	0.477	0.869**	0.606	-0.128	0.499
Density_Pyr_	0.474	0.249	0.07	0.331	-0.487	-0.489
Density_Xan_	-0.147	-0.125	-0.493	0.278	0.231	0.027
Density_Cry_	-0.403	-0.172	-0.11	0.437	-0.238	-0.001
Density_Bac_	-0.145	-0.353	0.746*	0.443	-0.168	0.158
Density_Chl_	-0.46	-0.278	0.098	0.568	-0.222	0.291
Biomass_TPh_	0.399	0.23	-0.169	0.503	-0.175	0.201
Biomass_Cya_	0.342	-0.391	0.323	0.6	-0.149	0.294
Biomass_Eug_	0.815**	0.837**	0.622	0.606	-0.128	0.499
Biomass_Pyr_	0.472	0.25	0.066	0.19	-0.331	-0.303
Biomass_Xan_	-0.147	-0.125	-0.492	0.286	0.226	0.033
Biomass_Cry_	-0.262	-0.08	0.107	0.412	-0.281	-0.238
Biomass_Bac_	-0.133	-0.355	-0.573	0.443	-0.168	0.158
Biomass_Chl_	-0.46	-0.279	0.097	0.568	-0.223	0.291

WT = water temperature; TN = total nitrogen; TP = total phosphorus; TPh = total phytoplankton; Cya = Cyanophyta; Eug = Euglenophyta; Pyr = Pyrrophyta; Xan = Xanthophyta; Cry = Cryptophyta; Bac = Bacillariophyta; Chl = Chlorophyta

*p<0.05

**p<0.01.

**Table 3:** The Pearson correlations between water temperature, total phosphorus, total nitrogen and phytoplankton of Dongping Lake in different seasons.

	Spring			Summer		
	WT	TN	TP	WT	TN	TP
Density_TPh_	0.787*	0.559	-0.578	-0.153	0.185	0.972**
Density_Cya_	0.014	-0.043	-0.289	-0.226	0.231	0.897**
Density_Eug_	0.807*	0.912**	-0.272	-0.175	0.157	0.993**
Density_Pyr_	-0.518	-0.191	-0.03	-0.257	-0.22	0.011
Density_Xan_	0.773*	0.942**	-0.326	--	--	--
Density_Cry_	0.821*	0.895**	-0.242	-0.171	0.262	0.824*
Density_Bac_	0.887**	0.39	-0.553	-0.115	0.162	0.939**
Density_Chl_	0.359	0.395	-0.413	0.033	0.297	0.870**
Biomass_TPh_	0.850**	0.591	-0.581	-0.147	0.171	0.971**
Biomass_Cya_	0.013	-0.043	-0.288	-0.226	0.231	0.897**
Biomass_Eug_	0.807*	0.912**	-0.272	-0.175	0.157	0.993**
Biomass_Pyr_	-0.56	-0.22	0.028	-0.257	-0.22	0.011
Biomass_Xan_	0.772*	0.942**	-0.328	--	--	--
Biomass_Cry_	0.826*	0.891**	-0.243	-0.172	0.262	0.825*
Biomass_Bac_	0.887**	0.39	-0.553	-0.115	0.162	0.939**
Biomass_Chl_	0.359	0.395	-0.413	0.033	0.297	0.870**

WT = water temperature; TN = total nitrogen; TP = total phosphorus; TPh = total phytoplankton; Cya = Cyanophyta; Eug = Euglenophyta; Pyr = Pyrrophyta; Xan = Xanthophyta; Cry = Cryptophyta; Bac = Bacillariophyta; Chl = Chlorophyta

*p<0.05

**p<0.01.

The top-down effects of zooplankton on phytoplankton were unclear in both lakes. Phytoplankton were positively correlated with zooplankton in Nansi Lake, indicating possible bottom-up effects of phytoplankton on zooplankton (Table 4). However, the top-down effects of zooplankton on phytoplankton were not significant in the two lakes. In Nansi Lake, Cyanophyta strongly regulated zooplankton in spring, and Cryptophyta did so in summer. During the seasonal changes, the density of Bacillariophyta in spring was positively correlated with zooplankton in summer. In Dongping Lake, the regulation of phytoplankton on zooplankton mainly occurred between Pyrrophyta and zooplankton (Table 5). In particular, the analyses indicated a significant relationship between Pyrrophyta and Crustacea. Most phytoplankton taxa variables in spring had a positive influence on the total density of zooplankton in summer. The results showed that the biomass accumulation of zooplankton and Crustacea mainly depended on phytoplankton to provide rich sources of food in spring, while the predation of zooplankton and Crustacea had weak top-down control on phytoplankton.

**Table 4:** The Pearson correlations between phytoplankton and zooplankton variables of Nansi Lake in different seasons.

		Spring				Summer			
		Density_TZ_	Density_Cru_	Biomass_TZ_	Biomass_Cru_	Density_TZ_	Density_Cru_	Biomass_TZ_	Biomass_Cru_
Spring	Density_Cya_	0.650	0.805**	0.815**	0.805**	0.757*	0.252	0.261	0.248
	Density_Bac_	-0.143	0.446	0.426	0.446	0.295	0.789*	0.788*	0.790*
	Biomass_Cya_	0.660	0.803**	0.814**	0.803**	0.755*	0.242	0.251	0.239
Summer	Density_Cry_	0.065	0.771*	0.751*	0.771*	0.488	0.730*	0.731*	0.725*
	Biomass_Cry_	0.099	0.685*	0.670*	0.685*	0.503	0.665	0.667*	0.660

TZ = total zooplankton; Cru = Crustacea; Cya = Cyanophyta; Cry = Cryptophyta; Bac = Bacillariophyta

*p<0.05

**p<0.01; Complete data is attached in Supporting information.

**Table 5:** The Pearson correlations between phytoplankton and zooplankton variables of Dongping Lake in different seasons.

		Spring				Summer			
		Density_TZ_	Density_Cru_	Biomass_TZ_	Biomass_Cru_	Density_TZ_	Density_Cru_	Biomass_TZ_	Biomass_Cru_
Spring	Density_TPh_	-0.051	-0.371	-0.026	-0.032	0.835**	-0.317	0.347	-0.246
	Density_Eug_	-0.160	-0.122	-0.206	-0.259	0.853**	-0.160	0.486	-0.086
	Density_Pyr_	-0.339	0.895**	-0.229	0.351	-0.419	-0.230	-0.468	-0.260
	Density_Xan_	-0.278	-0.001	-0.228	-0.003	0.728*	-0.260	0.295	-0.224
	Density_Cry_	-0.095	-0.220	-0.108	-0.171	0.877**	-0.149	0.561	-0.013
	Density_Bac_	0.322	-0.261	0.336	-0.079	0.812*	-0.032	0.401	-0.147
	Biomass_TPh_	-0.005	-0.355	-0.010	-0.119	0.886**	-0.261	0.396	-0.224
	Biomass_Eug_	-0.160	-0.122	-0.206	-0.259	0.853**	-0.160	0.486	-0.086
	Biomass_Pyr_	-0.331	0.857**	-0.225	0.314	-0.416	-0.256	-0.484	-0.282
	Biomass_Xan_	-0.280	-0.001	-0.226	0.007	0.725*	-0.263	0.290	-0.227
	Biomass_Cry_	-0.086	-0.227	-0.103	-0.181	0.885**	-0.147	0.566	-0.013
	Biomass_Bac_	0.322	-0.262	0.336	-0.079	0.812*	-0.032	0.402	-0.147
Summer	Density_Pyr_	0.588	-0.366	0.450	-0.371	0.036	0.859**	0.779*	0.961**
	Biomass_Pyr_	0.450	-0.371	0.588	-0.366	0.035	0.859**	0.779*	0.962**

TZ = total zooplankton; Cru = Crustacea; TPh = total phytoplankton; Eug = Euglenophyta; Pyr = Pyrrophyta; Xan = Xanthophyta; Cry = Cryptophyta; Bac = Bacillariophyta

*p<0.05

**p<0.01; Complete data is attached in Supporting information.

## Discussion

### The response of phytoplankton to bottom-up and top-down effects

Our study showed that the bottom-up effects of physical-chemical variables on phytoplankton were stronger than the top-down effects of zooplankton in both Nansi and Dongping lakes, which is consistent with previous studies [17,18,26]. The growth and reproduction of phytoplankton were influenced by physical-chemical factors in the water [27,28], among which water temperature and nutrient concentration contributed more than the other factors. The rate of photosynthesis of phytoplankton is probably promoted by the rising water temperature, which promotes the accumulation of biomass. In addition, seasonal shifts provide suitable living conditions for phytoplankton, such as promoting the absorption of nutrients at night by phytoplankton due to the increased daily minimum temperature in summer [29,30]. Nitrogen and phosphorus are essential nutrients for the growth of phytoplankton, and at a particular concentration, they can also limit the growth of phytoplankton [31,32]. In an applicable concentration range of nitrogen and phosphorus, increasing concentrations can promote phytoplankton growth [33,34]. Therefore, nutrients factors in water bodies can effectively control the density and biomass of phytoplankton through bottom-up effects.

Zooplankton, especially Crustacea, can directly affect the density and biomass of phytoplankton through predation, while zooplankton can also be influenced by fish predation. Previous studies have indicated that zooplankton are mainly controlled by top-down effects [35], and they deliver top-down effects on phytoplankton through food chains. The predation pressure of fish on zooplankton changes the biomass and density of zooplankton, which further influences the top-down effects of zooplankton on phytoplankton. Fish that feed on zooplankton prefer large Cladocera, followed by Copepods, which have a strong ability to escape, and finally, the smaller species of Rotifera [36,37]. In lakes with aquaculture, the community structure of Crustacea is mainly influenced by predation by fish, which is not significant enough to affect the biomass of phytoplankton [38]. Cage aquaculture in Nansi and Dongping lakes increased the feeding pressure on zooplankton due to the increasing fish stock, resulting in less zooplankton, especially Crustacea. However, the reduced zooplankton density in our study did not show measurable top-down effects of zooplankton on phytoplankton. Although some studies have shown that phytoplankton were under the top-down regulation of zooplankton, these studies were normally conducted in eutrophic ecosystems, such as eutrophic reservoirs, lakes, and bays [19,39]. Under field studies on bottom-up and top-down effects, the environmental complexity obscured the effects between phytoplankton and zooplankton with the participation of higher-level predators.

### Seasonal patterns of bottom-up and top-down effects

The bottom-up effects of physical-chemical variables were stronger than the top-down effects of zooplankton on phytoplankton in both Nansi and Dongping lakes in spring and summer. However, the seasonal bottom-up effects of physical-chemical factors and the top-down effects of zooplankton on phytoplankton were different between Nansi and Dongping lakes. The results showed a significant positive correlation between phytoplankton and physical-chemical factors and no significant negative correlation between phytoplankton and zooplankton in either lake. However, there was a strong correlation showing the bottom-up effects of physical-chemical on phytoplankton. In Nansi Lake, the bottom-up effects of physical-chemical on phytoplankton were stronger in spring than in summer, as there was no significant correlation in summer. The density and biomass of phytoplankton in summer increased significantly as nutrient concentrations and water temperature increased. The same conclusions can be drawn from Dongping Lake. Our results implied that the bottom-up effects of nutrients on phytoplankton also changed between seasons. The control of bottom-up effects on phytoplankton altered between nitrogen and phosphorus in Dongping Lake. Chai et al. [40] and Zhou and Liu [41] also found that these nutrient elements changed, which affected phytoplankton between seasons. The primary nutrients, which influenced phytoplankton in Dongping Lake, changed from TN in spring to TP in summer. The growth of phytoplankton needs a suitable N/P ratio [42], and a previous study found that the N/P ratios in Dongping Lake changed from 5.17 to 9.64 [43].

Frau et al. [44] pointed out that the control patterns of bottom-up and top-down effects on phytoplankton were different during different hydrological periods. The top-down effects on phytoplankton were stronger in the dry season, while the bottom-up effects were stronger in the wet season. Neither sunlight nor nutrients are limiting factors of phytoplankton biomass in summer, and it follows that a short-term top-down effect appears in the lake [45]. In Nansi and Dongping lakes, fish culture influenced the density of Crustacea through predation pressure, which led to no measurable top-down control of zooplankton on phytoplankton. However, the bottom-up effects of phytoplankton on zooplankton were significant in spring and summer. The growth of zooplankton biomass can also be affected by the biomass of phytoplankton due to predator-prey relationships [46]. As temperature rises in summer, higher predation pressure of fish leads to a decrease in the density of Daphnia [47,48], which in turn results in a decreased intensity of predation on phytoplankton. Our results showed reductions in both Cladocera and Copepoda, leading to weak top-down effects of zooplankton on phytoplankton in summer.

Contrary to expectations, the zooplankton did not significantly decrease the density and biomass of phytoplankton, which corresponds with a previous study [49]. Gliwicz [50] indicated that fish play an important role in the composition and distribution of the zooplankton community, which indirectly influences the top-down effects of zooplankton on phytoplankton. Fish culture blocks the top-down effects of zooplankton on phytoplankton in these kinds of aquatic ecosystems. Von Ruckert and Giani [51] suggested that fish regulate phytoplankton more than zooplankton in certain systems. As the phytoplankton composition changed from spring to summer and there were no significant top-down effects of zooplankton on phytoplankton, we also suggest the plant defense hypothesis, which states that herbivores control the plant species composition rather than plant biomass [49,52,53]. The diversity of phytoplankton possibly made the phytoplankton resilient to predation by zooplankton and fish, with changes in composition but not biomass. The top-down effects of zooplankton on phytoplankton were not significant, but they might contribute to shaping the community composition of phytoplankton [54–56].

## Conclusions

Our study showed that the bottom-up effects of physical-chemical on phytoplankton were weaker in summer than in spring in Nansi Lake, and there was a shift in the bottom-up effects of nutrients on phytoplankton in Dongping Lake. The control nutrients of bottom-up effects on phytoplankton altered from TN in spring to TP in summer. While the bottom-up effects of physical-chemical on phytoplankton are clear, the top-down effects of zooplankton on phytoplankton, which may have been regulated indirectly by fish, are more difficult to predict in aquatic management. Conclusions should be drawn cautiously because more data are needed for a thorough analysis of the two effects. Our results can serve as a basis for identifying how phytoplankton are influenced, which have implications for developing sustainable management strategies and conserving services in freshwater lake ecosystems.

## Supporting information

S1 Table The Pearson correlations between phytoplankton and zooplankton variables of Nansi Lake in different seasons. (DOCX)

S2 Table The Pearson correlations between phytoplankton and zooplankton variables of Dongping Lake in different seasons. (DOCX)
