# Supplementary material for: Bottom-up and top-down effects on phytoplankton communities in two freshwater lakes
Source: PLoS One. 2020 Apr 9;15(4):e0231357. doi: 10.1371/journal.pone.0231357 (PMC7145108; doi:10.1371/journal.pone.0231357)
Supplement: S1 Table — (DOCX) [file pone.0231357.s001.docx]

**S1 Table. The Pearson correlations between phytoplankton and zooplankton variables of Nansi Lake in different seasons.**

|  |  | Spring | | | | Summer | | | |
| --- | --- | --- | --- | --- | --- | --- | --- | --- | --- |
|  |  | Density_TZ_ | Density_Cru_ | Biomass_TZ_ | Biomass_Cru_ | Density_TZ_ | Density_Cru_ | Biomass_TZ_ | Biomass_Cru_ |
| Spring | Density_TPh_ | -.261 | -.373 | -.376 | -.373 | -.344 | -.142 | -.146 | -.145 |
|  | Density_Cya_ | .650 | **.805^**^** | **.815^**^** | **.805^**^** | **.757^*^** | .252 | .261 | .248 |
|  | Density_Eug_ | -.026 | -.114 | -.112 | -.114 | -.023 | -.298 | -.296 | -.299 |
|  | Density_Pyr_ | .403 | -.045 | -.022 | -.045 | .379 | .011 | .021 | .017 |
|  | Density_Xan_ | -.157 | -.363 | -.361 | -.363 | .040 | .041 | .045 | .047 |
|  | Density_Cry_ | -.171 | -.441 | -.437 | -.441 | -.398 | -.320 | -.325 | -.323 |
|  | Density_Bac_ | -.143 | .446 | .426 | .446 | .295 | **.789^*^** | **.788^*^** | **.790^*^** |
|  | Density_Chl_ | -.529 | .040 | .010 | .040 | -.180 | .227 | .224 | .222 |
|  | Biomass_TPh_ | .014 | .412 | .401 | .412 | .078 | .529 | .521 | .527 |
|  | Biomass_Cya_ | .660 | **.803^**^** | **.814^**^** | **.803^**^** | **.755^*^** | .242 | .251 | .239 |
|  | Biomass_Eug_ | .291 | -.004 | .012 | -.004 | -.107 | -.216 | -.219 | -.216 |
|  | Biomass_Pyr_ | .400 | -.050 | -.028 | -.050 | .372 | .008 | .018 | .014 |
|  | Biomass_Xan_ | -.157 | -.364 | -.361 | -.364 | .040 | .041 | .044 | .047 |
|  | Biomass_Cry_ | .070 | -.270 | -.258 | -.270 | .003 | -.212 | -.212 | -.213 |
|  | Biomass_Bac_ | -.131 | .572 | .548 | .572 | .130 | .666 | .660 | .663 |
|  | Biomass_Chl_ | -.529 | .040 | .010 | .040 | -.181 | .227 | .224 | .222 |
| Summer | Density_TPh_ | -.460 | -.274 | -.290 | -.274 | -.196 | -.231 | -.228 | -.231 |
|  | Density_Cya_ | -.526 | -.414 | -.430 | -.414 | -.424 | -.305 | -.304 | -.305 |
|  | Density_Eug_ | .295 | -.057 | -.040 | -.057 | -.134 | -.369 | -.366 | -.371 |
|  | Density_Pyr_ | -.558 | -.003 | -.033 | -.003 | -.096 | .275 | .271 | .271 |
|  | Density_Xan_ | -.357 | -.222 | -.234 | -.222 | -.157 | -.229 | -.229 | -.230 |
|  | Density_Cry_ | .065 | **.771^*^** | **.751^*^** | **.771^*^** | .488 | **.730^*^** | **.731^*^** | **.725^*^** |
|  | Density_Bac_ | -.405 | -.227 | -.243 | -.228 | -.086 | -.214 | -.210 | -.215 |
|  | Density_Chl_ | -.453 | -.326 | -.341 | -.326 | -.288 | -.319 | -.317 | -.320 |
|  | Biomass_TPh_ | -.447 | -.278 | -.294 | -.278 | -.173 | -.245 | -.241 | -.245 |
|  | Biomass_Cya_ | -.526 | -.414 | -.430 | -.414 | -.425 | -.305 | -.304 | -.305 |
|  | Biomass_Eug_ | .295 | -.057 | -.040 | -.057 | -.134 | -.369 | -.366 | -.371 |
|  | Biomass_Pyr_ | -.300 | .507 | .476 | .507 | .063 | .576 | .571 | .574 |
|  | Biomass_Xan_ | -.361 | -.227 | -.240 | -.227 | -.163 | -.233 | -.233 | -.235 |
|  | Biomass_Cry_ | .099 | **.685^*^** | **.670^*^** | **.685^*^** | .503 | .665 | **.667^*^** | .660 |
|  | Biomass_Bac_ | -.405 | -.227 | -.242 | -.227 | -.086 | -.214 | -.210 | -.215 |
|  | Biomass_Chl_ | -.453 | -.326 | -.341 | -.326 | -.288 | -.319 | -.317 | -.320 |

TZ = total zooplankton; Cru = Crustacea; TPh = total phytoplankton; Cya = Cyanophyta; Eug = Euglenophyta; Pyr = Pyrrophyta; Xan = Xanthophyta; Cry = Cryptophyta; Bac = Bacillariophyta; Chl = Chlorophyta; *p<0.05; **p<0.01.
