# Supplementary material for: Bottom-up and top-down effects on phytoplankton communities in two freshwater lakes
Source: PLoS One. 2020 Apr 9;15(4):e0231357. doi: 10.1371/journal.pone.0231357 (PMC7145108; doi:10.1371/journal.pone.0231357)
Supplement: S2 Table — (DOCX) [file pone.0231357.s002.docx]

**S2 Table. The Pearson correlations between phytoplankton and zooplankton variables of Dongping Lake in different seasons.**

|  |  | Spring | | | | Summer | | | |
| --- | --- | --- | --- | --- | --- | --- | --- | --- | --- |
|  |  | Density_TZ_ | Density_Cru_ | Biomass_TZ_ | Biomass_Cru_ | Density_TZ_ | Density_Cru_ | Biomass_TZ_ | Biomass_Cru_ |
| Spring | Density_TPh_ | -.051 | -.371 | -.026 | -.032 | **.835^**^** | -.317 | .347 | -.246 |
|  | Density_Cya_ | -.156 | -.045 | .105 | .671 | -.035 | -.400 | -.295 | -.346 |
|  | Density_Eug_ | -.160 | -.122 | -.206 | -.259 | **.853^**^** | -.160 | .486 | -.086 |
|  | Density_Pyr_ | -.339 | **.895^**^** | -.229 | .351 | -.419 | -.230 | -.468 | -.260 |
|  | Density_Xan_ | -.278 | -.001 | -.228 | -.003 | **.728^*^** | -.260 | .295 | -.224 |
|  | Density_Cry_ | -.095 | -.220 | -.108 | -.171 | **.877^**^** | -.149 | .561 | -.013 |
|  | Density_Bac_ | .322 | -.261 | .336 | -.079 | **.812^*^** | -.032 | .401 | -.147 |
|  | Density_Chl_ | -.365 | -.487 | -.509 | -.383 | .590 | -.385 | .262 | -.165 |
|  | Biomass_TPh_ | -.005 | -.355 | -.010 | -.119 | **.886^**^** | -.261 | .396 | -.224 |
|  | Biomass_Cya_ | -.155 | -.045 | .106 | .671 | -.035 | -.400 | -.295 | -.346 |
|  | Biomass_Eug_ | -.160 | -.122 | -.206 | -.259 | **.853^**^** | -.160 | .486 | -.086 |
|  | Biomass_Pyr_ | -.331 | **.857^**^** | -.225 | .314 | -.416 | -.256 | -.484 | -.282 |
|  | Biomass_Xan_ | -.280 | -.001 | -.226 | .007 | **.725^*^** | -.263 | .290 | -.227 |
|  | Biomass_Cry_ | -.086 | -.227 | -.103 | -.181 | **.885^**^** | -.147 | .566 | -.013 |
|  | Biomass_Bac_ | .322 | -.262 | .336 | -.079 | **.812^*^** | -.032 | .402 | -.147 |
|  | Biomass_Chl_ | -.365 | -.487 | -.509 | -.383 | .590 | -.385 | .262 | -.165 |
| Summer | Density_TPh_ | .054 | -.252 | .131 | .210 | .236 | -.269 | .108 | -.069 |
|  | Density_Cya_ | .106 | -.260 | .231 | .340 | .031 | -.129 | .073 | .060 |
|  | Density_Eug_ | .159 | -.426 | .182 | .030 | .445 | -.119 | .389 | .118 |
|  | Density_Pyr_ | .588 | -.366 | .450 | -.371 | .036 | **.859^**^** | **.779^*^** | **.961^**^** |
|  | Density_Xan_ | .^b^ | .^b^ | .^b^ | .^b^ | .^b^ | .^b^ | .^b^ | .^b^ |
|  | Density_Cry_ | .220 | -.350 | .339 | .296 | .151 | .099 | .316 | .273 |
|  | Density_Bac_ | -.012 | -.212 | .041 | .150 | .298 | -.381 | .050 | -.194 |
|  | Density_Chl_ | -.114 | -.045 | .086 | .539 | .082 | -.387 | -.137 | -.251 |
|  | Biomass_TPh_ | .131 | .210 | .050 | -.250 | .261 | -.283 | .114 | -.082 |
|  | Biomass_Cya_ | .231 | .340 | .106 | -.260 | .031 | -.128 | .073 | .060 |
|  | Biomass_Eug_ | .182 | .030 | .159 | -.426 | .445 | -.119 | .389 | .118 |
|  | Biomass_Pyr_ | .450 | -.371 | .588 | -.366 | .035 | **.859^**^** | **.779^*^** | **.962^**^** |
|  | Biomass_Xan_ | .^b^ | .^b^ | .^a^ | .^a^ | .^a^ | .^a^ | .^b^ | .^b^ |
|  | Biomass_Cry_ | .339 | .296 | .220 | -.351 | .150 | .099 | .317 | .274 |
|  | Biomass_Bac_ | .041 | .150 | -.012 | -.212 | .298 | -.381 | .050 | -.194 |
|  | Biomass_Chl_ | .086 | .539 | -.114 | -.045 | .082 | -.387 | -.137 | -.251 |

TZ = total zooplankton; Cru = Crustacea; TPh = total phytoplankton; Cya = Cyanophyta; Eug = Euglenophyta; Pyr = Pyrrophyta; Xan = Xanthophyta; Cry = Cryptophyta; Bac = Bacillariophyta; Chl = Chlorophyta; *p<0.05; **p<0.01.
